# Glycyrrhizic Acid Can Attenuate Metabolic Deviations Caused by a High-Sucrose Diet without Causing Water Retention in Male Sprague-Dawley Rats

**DOI:** 10.3390/nu6114856

**Published:** 2014-11-04

**Authors:** Hamish Alexander Fernando, Chanchal Chandramouli, Dayang Rosli, Yi Lyn Lam, Sheau Ting Yong, Hui Ping Yaw, So Ha Ton, Khalid Abdul Kadir, Amanda Sainsbury

**Affiliations:** 1 School of Science, Monash University Sunway Campus, Jalan Lagoon Selatan, Bandar Sunway, Selangor Darul Ehsan 46150, Malaysia; E-Mails: c.chandramouli@student.unimelb.edu.au (C.C.); dayangrosli@live.com (D.R.); springbreeze88@hotmail.com (Y.L.L.); crystaljacy@gmail.com (S.T.Y.); hpyaw1@student.monash.edu (H.P.Y.); ton.so.ha@monash.edu (S.H.T.); 2 School of Medicine and Health Sciences, Monash University Sunway Campus, Jalan Lagoon Selatan, Bandar Sunway, Selangor Darul Ehsan 46150, Malaysia; E-Mail: khalid.kadir@monash.edu; 3 The Boden Institute of Obesity, Nutrition, Exercise and Eating Disorders, Sydney Medical School, The University of Sydney, Camperdown, New South Wales 2006, Australia; E-Mail: amanda.salis@sydney.edu.au

**Keywords:** glycyrrhizic acid, metabolic syndrome, high-sucrose diet, 11β-HSD2, side effects, edema, angiotensin II, electrolytes

## Abstract

Glycyrrhizic acid (GA) ameliorates many components of the metabolic syndrome, but its potential therapeutic use is marred by edema caused by inhibition of renal 11β-hydroxysteroid dehydrogenase 2 (11β-HSD2). We assessed whether 100 mg/kg per day GA administered orally could promote metabolic benefits without causing edema in rats fed on a high-sucrose diet. Groups of eight male rats were fed on one of three diets for 28 days: normal diet, a high-sucrose diet, or a high-sucrose diet supplemented with GA. Rats were then culled and renal 11β-HSD2 activity, as well as serum sodium, potassium, angiotensin II and leptin levels were determined. Histological analyses were performed to assess changes in adipocyte size in visceral and subcutaneous depots, as well as hepatic and renal tissue morphology. This dosing paradigm of GA attenuated the increases in serum leptin levels and visceral, but not subcutaneous adipocyte size caused by the high-sucrose diet. Although GA decreased renal 11β-HSD2 activity, it did not affect serum electrolyte or angiotensin II levels, indicating no onset of edema. Furthermore, there were no apparent morphological changes in the liver or kidney, indicating no toxicity. In conclusion, it is possible to reap metabolic benefits of GA without edema using the current dosage and treatment time.

## 1. Introduction

Metabolic syndrome is a breakdown of various metabolic functions resulting in abnormalities that pose a significant risk for cardiovascular disease and type II diabetes mellitus [1]. The characteristic aberrations of the metabolic syndrome include central obesity, hyperglycaemia, hypertension, insulin resistance and dyslipidaemia [2]. Over the past decades, decreased exercise due to improvements in technology, combined with the spread of westernized diets, have caused a significant increase in the incidence of metabolic syndrome in developed and developing countries alike [2]. Westernized diets include primarily those high in energy content, from both fat and sucrose. High-sucrose feeding alone has been shown to trigger many of the abnormalities of the metabolic syndrome [3]. Although there are several drugs available to treat the components of the metabolic syndrome, many of those that are commonly available tend to be expensive—particularly for people in developing countries—and/or associated with numerous side-effects [4,5,6,7]. Hence the search for alternate substances that could prevent/reverse the onset and progression of the metabolic syndrome has become increasingly important.

One potential treatment for the metabolic syndrome is glycyrrhizic acid (GA), which is the primary bioactive constituent of licorice, obtained from the root of the *Glycyrrhiza glabra* shrub [8]. Our research has indicated various benefits of GA consumption in rats fed on either a high-fat [9] or high sucrose diet (HSD) [3]. These benefits include reductions in circulating concentrations of glucose and insulin, enhanced insulin sensitivity, improved circulating lipid profiles, and favorable alterations in the expression of genes and activity of enzymes involved in lipid metabolism and energy balance [3,9,10]. The dose of GA required to bring about metabolic benefits depends on the duration and route of administration, as previously reviewed [11].

The potential of GA to treat the metabolic syndrome stems primarily from its ability to inhibit the enzyme 11β-hydroxysteroid dehydrogenase 1 (11β-HSD1) [11]. However, this action is non-selective, as GA also inhibits the other isoform of this enzyme, 11β-HSD2 [11]. The two isoforms of 11β-HSD have been shown to have opposing functions, which is of vital consequence with regards to the effects of GA consumption. 11β-HSD2 is a dehydrogenase that catalyzes the reversible conversion of active glucocorticoids (corticosterone in rats, cortisol in humans) to their inactive derivatives (11β-dehydrocorticosterone in rats, cortisone in humans) [12]. On the other hand, 11β-HSD1 can act both as a dehydrogenase, to catalyze the deactivation of glucocorticoids, as well as a reductase, to catalyze the activation of glucocorticoids [12,13]. In intact cells, the reductase actions of 11β-HSD1 that result in glucocorticoid activation have been shown to be more potent than their dehydrogenase actions [12].

As seen from the above, inhibition of 11β-HSD1 by GA will be expected to result in increased production of inactive glucocorticoids relative to active glucocorticoids. This brings about many of the benefits of GA with regards to the metabolic syndrome [3,9]. However, the concurrent inhibition of 11β-HSD2 by GA is potentially detrimental, as it could lead to edema, hypertension and hypokalemia, all of which have been observed as side effects in some instances of GA administration in humans and animals [11,14]. This is because the expression of 11β-HSD2 is highest in tissues that are targeted significantly by aldosterone, such as the kidneys, which have an abundance of mineralocorticoid receptors [12]. As the affinity of mineralocorticoid receptors for glucocorticoids and aldosterone is similar [14], the presence of higher concentrations of active glucocorticoids—as occurs with GA-induced suppression of 11β-HSD 2 [14]—could lead to their competitive binding to mineralocorticoid receptors, leading to a syndrome of apparent mineralocorticoid excess [14,15,16]. This is presented in the form of electrolyte imbalances, which are responsible for the afore-mentioned side effects [14]. This can also depress the renin-angiotensin aldosterone system (RAAS) and increase the levels of atrial natriuretic peptide in order to compensate for the changes in water balance caused by fluctuations in electrolyte levels [17]. Furthermore, in some cases, these side effects could also be brought about by the action of glycyrrhetinic acid, the primary metabolite of GA, via direct action on mineralocorticoid receptors [18,19]. Initial manifestation of GA-induced side effects are generally associated with 11β-HSD2 suppression, while side effects caused by direct effects of glycyrrhetinic acid are evident when serum concentrations of the latter are consistent with its affinity for mineralocorticoid receptors [19]. It is for the above reasons that, despite its known metabolic benefits as shown in animal models, GA is not yet a viable treatment option for disorders of the metabolic syndrome. The side effects of GA vary depending on the route of administration [11]. For example, while topical GA application significantly reduced subcutaneous fat levels in healthy women with no change in blood pressure or circulating renin activity, aldosterone or cortisol levels [20], oral GA administration to healthy men and women caused significant fat loss along with water retention, thereby leading to no significant reduction in the body-mass index [21]. In addition, the side effects of GA consumption are clearly dose-dependent, with lower doses inducing lesser, or no side effects relative to higher doses [22]. As in the case of metabolic benefits, the lowest dose at which side effects appear to manifest is dependent upon the duration and route of administration. For example, the toxicity of a given dose of GA is greater when administered for longer durations, and when administered subcutaneously or intraperitoneally, compared to topical or oral administrations [11].

Metabolic benefits of GA on lipid metabolism in rats on a normal chow diet were apparent at doses as low as 50 mg/kg per day for one week [10]. Unpublished data from our lab, however, indicated that GA at 100 mg/kg per day for four weeks was more effective at improving metabolic parameters, especially in rats exhibiting components of the metabolic syndrome due to consumption of high-fat or high sucrose diets. Since we have established many benefits of GA consumption with regards to the metabolic syndrome at a dose of 100 mg/kg per day for four weeks in rats fed on a HSD [3], it is important to assess how this dosage fares in terms of potential side effects. With such information we could establish whether it would be possible to reap the favorable effects of GA while minimizing detrimental effects, thus increasing its viability as a treatment option for the metabolic syndrome.

A previous study from our team showed that oral GA administration at 100 mg/kg per day for four weeks to rats on a HSD induced metabolic benefits without any increase in blood pressure, albeit blood pressure remained significantly greater than that of control rats on a normal chow diet in both HSD and HSD + GA groups [3]. This indicated that high-sucrose feeding increased blood pressure relative to control levels, while oral GA administration at this dose and duration did not exacerbate diet-induced hypertension. However, the possible onset of early precursors to hypertension, such as hypokalemia (a deficiency of potassium in the bloodstream) and edema, were not examined in that study. In the current study, performed over the same duration and at the same dosage in high sucrose-fed rats, we thus examined kidney 11β-HSD2 activity levels, as its inhibition could cause many of the side effects of GA, as discussed above. Serum electrolyte (Na^+^, K^+^) levels were also examined, as changes in their concentrations are associated with edema [11,14], as well as the possibility of the eventual development of hypertension [11,14]. Furthermore, as depression of the RAAS is observable in cases of mineralocorticoid excess syndrome [17], we also analyzed our GA-treated sucrose-fed rats for serum levels of angiotensin II, which is responsible for the activation of aldosterone. Potential toxic effects of GA consumption at the current dose and duration of administration on the liver and kidney were also examined by histological analyses. Finally, we analyzed how GA at this dosage would affect food intake, circulating leptin levels, and adipocyte size in both subcutaneous and visceral depots in sucrose-fed rats. As leptin is a major regulator of energy homeostasis, assessing the effects of GA on leptin along with adipocyte size may give further clues on the overall mechanism of action of GA with regard to metabolic benefits.

## 2. Experimental Section

### 2.1. Treatment of Rats and Sample Collection

#### 2.1.1. Animal Ethics

Approval to use rats for the purpose of this experiment was obtained from the Monash University School of Biomedical Science Animal Ethics Committee (approval number: MARP/2012/043; approval date: 8 May 2012), according to the 2004 Australian Code of Practice for the Care and Use of Animals for Scientific Purposes and the Monash University Animal Welfare Committee Guidelines and Policies (Prevention of Cruelty to Animals Act 1986).

#### 2.1.2. Animal Preparation

Twenty-four male Sprague Dawley rats of approximately 180 g–220 g (~7 weeks old) were purchased from the animal breeding facility of Monash University Sunway Campus, Malaysia. Only male rats were included in the study cohort to avoid any effects of cyclical changes in hormones in female rats. Rats were 6–8 weeks old upon commencement of the study and housed individually in polypropylene cages of approximately 35 × 25 × 20 cm. The cages contained shredded paper bedding.

#### 2.1.3. Experimental Design

The 24 rats were divided randomly into three equal groups: Chow, High Sucrose Diet (HSD) and HSD + GA. Rats in the Chow group were fed standard rat chow (Gold Coin, Selangor, Malaysia). The HSD was prepared by mixing powdered rat chow with cane sugar (Gula Prai, Prai, Malaysia) at a 2:3 ratio. Ten milliliter of water was added to the mixture, which was mixed to form a thick dough. Pellets were formed by hand and then baked at 160 °C for 5 min and then stored at 4 °C. All rats were given *ad libitum* access to food for the 28 days of the experiment, unless otherwise stated. The amount of food consumed over the past 24 h was evaluated at approximately 09:00 each day, and the average food consumption over the course of the treatment period was used to measure mean energy intake per day for each group. While rats in the Chow and HSD groups were given tap water to drink *ad libitum*, those in the HSD + GA group were given tap water with GA, designed to deliver 100 mg of GA per kg bodyweight per day based on average body weight and mean water consumption in the preceding week by the rats in that group. The GA-containing water was freshly prepared each week. All water bottles were topped to 250 mL every week, and the amount of water consumed over the past 24 h was evaluated at approximately 09:00 each day.

#### 2.1.4. Collection of Tissue Samples

At the end of 28 days on the 3 different diets, all 24 rats were fasted for 12 h and then anaesthetized with intraperitoneal sodium pentobarbital (150 mg/kg) between 10:00 and 11:00.

Using a 5 mL syringe with a 22 G needle, blood was drawn from the apex of the heart, left to stand for 20 min to clot at room temperature, and then centrifuged at 12,000× *g* for 10 min at 4 °C. The resulting serum was stored at −80 °C until needed.

The four tissues of interest, namely the subcutaneous and visceral adipose tissue (SAT and VAT), liver and kidney were harvested. Portions of the kidney samples from each animal were weighed, minced and placed immediately into separate 15 mL Falcon tubes containing ice-cold Krebs-Ringer bicarbonate (KRB) buffer (2 mL per g of tissue) for the measurement of 11β-HSD2 activities. Sections of the kidney, liver, SAT and VAT obtained from each animal were also washed, cut into approximately 5 × 5 × 5 mm pieces, and immersed in 10% neutral-buffered formalin for subsequent histological analysis.

### 2.2. Determination of 11β-Hydroxysteroid Dehydrogenase 2 Activity

Determination of 11β-HSD2 activity was conducted by quantifying the amount of 11-dehydrocorticosterone produced from externally administered corticosterone by the enzyme present in a fixed mass of kidney tissue.

Kidney tissues collected as described above were homogenized and then centrifuged at 4 °C and 14,000× *g* for 20 min. Protein concentrations in tissue homogenate supernatants were determined using the modified Lowry’s method. Supernatants containing 50 mg of tissue protein were mixed with the substrate mixture for 11-βHSD2, and then KRB buffer was used to top up the reaction mixture to 500 µL, giving a final concentration of 0.35 mmol/L NAD^+^, 0.1 mmol/L corticosterone, 0.2% glucose, 0.2% bovine serum albumin and 5% ethanol. Reaction mixtures were incubated for 1 h at 37 °C and were then immediately placed into a −20 °C freezer in order to terminate the reaction, and stored there until the next step. Next, 800 μL of ethyl acetate was pipetted into each microcentrifuge tube and tubes were mixed in an orbital shaker at 100 RPM for 30 min at room temperature. The samples were then centrifuged at 16,000× *g* and room temperature for 10 min. The upper (organic) layer of the centrifuged samples were separated and added to a new microcentrifuge tube, and ethyl acetate was evaporated using flowing nitrogen gas. Dried content was stored at −20 °C until quantification of glucocorticoids using high-performance liquid chromatography (HPLC). Reverse-phase chromatography was used to separate corticosterone (substrate) from 11-dehydrocorticosterone (product). Three-hundred microliter of HPLC mobile phase (20% methanol, 30% acetonitrile and 50% water v/v) was added to each microcentrifuge tube containing the dried glucocorticoid content. A series of cortisone (11-dehydrocorticosterone equivalent) standards were used to make a standard curve. A 100 µL Hamilton HPLC syringe was used to inject 30 µL of standard or sample into a 20 µL sample loop of the Perkin Elmer 200 Liquid Chromatography pump (Shelton, CT, USA). A Waters Symmetry^®^ C18 column (Tokyo, Japan) with dimensions 3.9 mm (diameter) × 150 mm (length) was used for separation of sample glucocorticoids. A linear methanol-acetonitrile-water gradient from 10:15:75 (v/v) to 20:30:50 (v/v) in the first 5 min was employed to separate the glucocorticoids. Isocratic elution then followed for the next 10 min. The constant flow rate of the mobile phase was set at 1.00 mL/min. Spectrophotometric absorbance was measured at 254 nm. Enzyme activity was expressed in units (U), in which 1 U of 11β-HSD2 activity is defined as one picomole of 11-dehydrocorticosterone produced per 50 mg of tissue protein used, per h of incubation at 37 °C.

### 2.3. Determination of Serum Angiotensin II and Leptin Levels

Serum angiotensin II and leptin levels were determined using the AGT/Agt (Human, Rat, Mouse) ELISA kit from Abnova (Taipei City, Taiwan) and the Millipore (Rat, Mouse) ELISA kit (Billerica, MA, USA) respectively, according to the manufacturers’ manuals.

### 2.4. Electrolyte Concentration Determination

Electrolyte concentrations in serum were determined using atomic absorption spectrophotometry and the Beer-Lambert law.

#### 2.4.1. Serum Sodium Ion Level Determination

A series of standard sodium solutions were used to make a standard curve. Blank and standard solutions were aspirated and measured with the Perkin Elmer Atomic Absorption spectrophotometer Analyst 100 (Waltham, MA, USA) using a sodium/potassium hollow cathode lamp at 589.0 nm.

Two-hundred microliter of serum was diluted to 100 mL with distilled water in a volumetric flask. These samples were then aspirated into the spectrophotometer and the concentration of sodium in the serum was calculated from comparison with the standard curve.

#### 2.4.2. Serum Potassium Ion Level Determination

The method to determine serum potassium levels was similar to that used to measure serum sodium levels, with the exception that the 200 µL serum was diluted in 10 mL of distilled water, and the wavelength used was 766.5 nm.

### 2.5. Histological Analyses

Analyses were conducted to examine liver and kidney tissue morphology and to assess the size of subcutaneous and visceral adipocytes. The haematoxylin and eosin stain (H & E stain) was used for these purposes. H & E-stained SAT and VAT sections were viewed using Olympus BX51 microscope (New York, NY, USA). The sizes of 25 adipocytes (µm^2^) per tissue section per field view for five views were measured using the Nikon Instruments-Element D 2.30 software (Nikon Instruments Inc., Melville, NY, USA).

### 2.6. Statistical Analysis

Statistical analysis of all data was performed using Statistical Package for the Social Sciences (SPSS) Version 16.0. Data distribution was analyzed using the Kolmogorov-Smirnov and Shapiro-Wilk tests. Data with parametric distribution were analyzed using Analysis of Variance (ANOVA). If the results were significant, a Post Hoc (Tukey’s) test was performed to identify differences. Non-parametric data were analyzed using the Kruskall-Wallis test. In all analyses, a *p* value ≤ 0.05 was considered to be statistically significant.

## 3. Results

### 3.1. Glycyrrhizic Acid Intake Ameliorated the Effects of a High-Sucrose Diet on Serum Leptin Levels and Adipocyte Size

**Figure 1:**
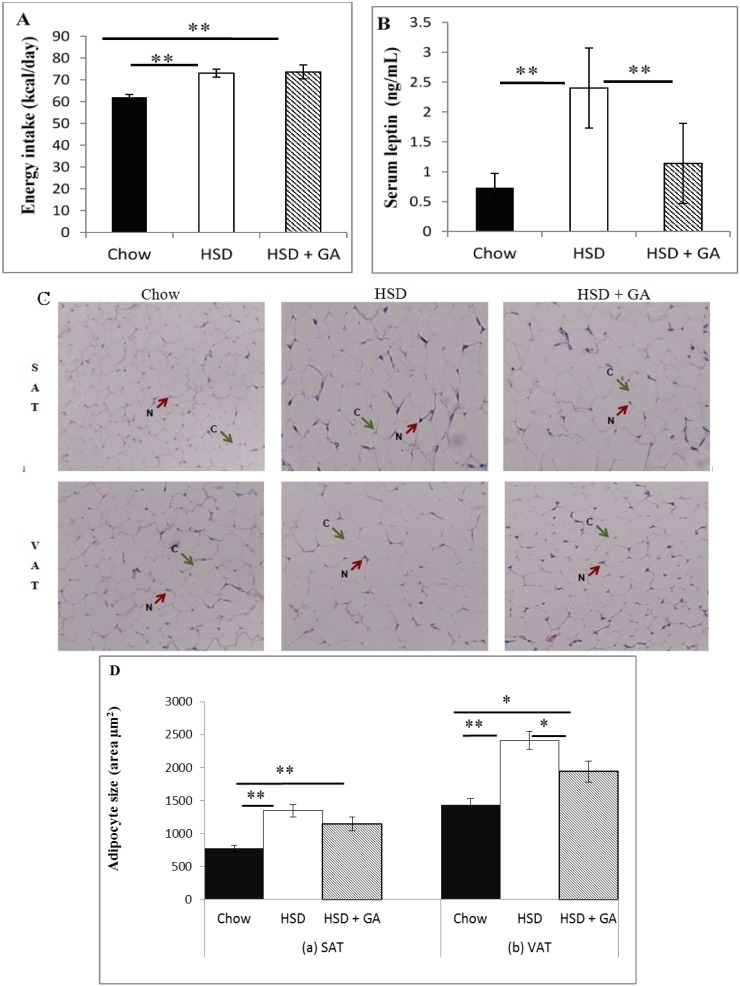
Glycyrrhizic acid consumption ameliorated effects of a high-sucrose diet on metabolic parameters. (**A**) Daily energy intake and (**B**) serum leptin concentrations, as well as (**C**) representative examples of haemotoxylin and eosin (H & E) stained sections of subcutaneous and visceral adipose tissue (SAT and VAT, 200× magnification) and (**D**) adipocyte size (cross-sectional area) in SAT and VAT depots from rats fed normal chow, a high-sucrose diet (HSD), or a HSD supplemented with 100 mg/kg per day glycyrrhizic acid (HSD + GA), administered orally for four weeks. One unit (U) of 11β-HSD2 activity is defined as one picomole of 11-dehydrocorticosterone produced per 50 mg of tissue protein used, per h of incubation at 37 °C. Data are means ± SEM of 8 male rats per group. * *p* ≤ 0.05, ** *p* ≤ 0.01 for the comparison shown by horizontal bars.

Our studies have previously shown that GA consumption attenuates the effects of a HSD to increase serum glucose and insulin levels [3]. Further metabolic effects of this dose of GA (100 mg/kg per day administered orally for four weeks) were assessed by examining effects on leptin levels and adipocyte size. Although GA did not attenuate the increased energy intake induced by the HSD (Figure 1A), it reduced serum leptin levels significantly (Figure 1B). GA intake also prevented adipocyte hypertrophy caused by the HSD in the visceral but not the subcutaneous adipose tissue depots, as shown in Figure 1C,D. These results, combined with those obtained by Chandramouli *et al*. [3], indicate the potential for significant modulative capabilities of GA at the current dose and duration, with regard to metabolic parameters.

### 3.2. Glycyrrhizic Acid Did Not Change Circulating Levels of Electrolytes or Renin Angiotensin II, or Alter Liver or Kidney Histomorphology, Despite Reducing Kidney 11β-HSD2 Activity

**Figure 2:**
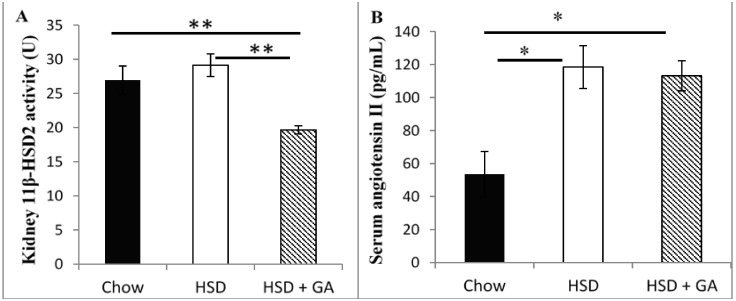

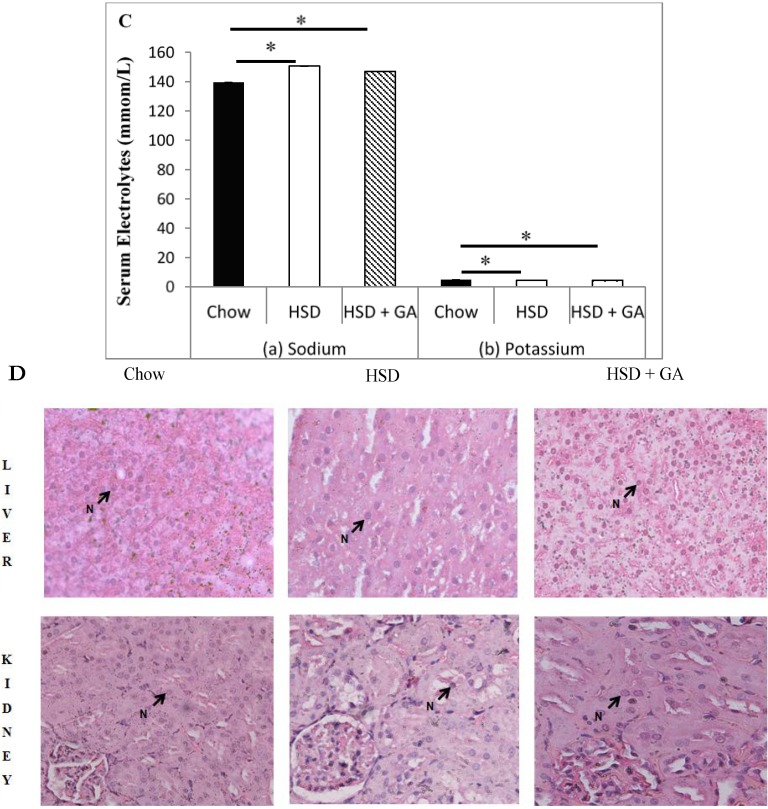
Glycyrrhizic acid consumed at the given dosage did not lead to signs of changes in fluid balance despite reduced kidney 11β-HSD2 activity. (**A**) Kidney 11β-HSD2 activity; (**B**) serum angiotensin II concentrations; (**C**) serum electrolyte concentrations ((a):sodium; (b):potassium), and (**D**) representative samples of haemotoxylin and eosin (H & E) stained sections of liver and kidney (×200 magnification) from rats fed normal chow, a high-sucrose diet (HSD), or a HSD supplemented with 100 mg/kg per day glycyrrhizic acid (HSD + GA), administered orally for four weeks. Data are means ± SEM of 8 male rats per group. * *p* ≤ 0.05, ** *p* ≤ 0.01 for the comparison shown by horizontal bars.

As mentioned in the introduction, the primary side effects of GA are hypertension and edema, which are caused by increased water retention resulting from electrolyte imbalances caused by inhibition of renal 11β-HSD2 activity [14]. Water retention also causes a compensatory decrease in the RAAS activity [17]. Although our dose of 100 mg/kg per day GA consumption for four weeks attenuated the effects of the HSD on metabolic parameters as described above, and although this dose of GA significantly decreased the activity of 11β-HSD2 in the kidney of rats in the HSD + GA group relative to that in the chow or high sucrose fed rats (Figure 2A), it did not result in electrolyte imbalances as indicated by alterations in serum concentrations of angiotensin II (Figure 2B) or the electrolytes sodium or potassium (Figure 2C), relative to rats fed on a HSD. This suggests that the current dose and treatment time of GA used in this study did not affect renal 11β-HSD2 activities significantly enough to alter these parameters over and above the effects of the HSD. Furthermore, haemotoxylin and eosin stained tissue sections showed no change in hepatic or kidney tissue morphology (Figure 2D), indicating no visible toxic effects on these organs during metabolism and excretion of GA.

## 4. Discussion

Despite its known therapeutic potential for metabolic disorders, GA consumption is still not used as a mainstream therapy for the metabolic syndrome due to its side effects of edema and hypertension. In this study, we found that a daily oral dosage of 100 mg/kg per day GA administered for four weeks significantly averted metabolic deviations associated with a HSD in rats without any concomitant indication of side effects associated with previous GA paradigms. Indeed, GA prevented the increases in adipocyte size and circulating leptin levels caused by a HSD, despite no effect on energy intake. No apparent changes in electrolyte balance (as indicated by serum sodium or potassium concentrations) or serum levels of angiotensin II were detected with GA consumed for the duration of this study at the current dose, despite a significant decrease in kidney 11β-HSD2 activity. Furthermore, no changes in liver or kidney histomorphology were apparent, suggesting no toxic effect of this dosing paradigm of GA on these tissues. These results suggest that by carefully modulating dosage and treatment time, GA could be a potentially safe therapeutic agent against metabolic changes associated with a HSD.

The inhibition of renal 11β-HSD2 activity by GA is known to be a primary cause of the undesirable side effects associated with GA intake [11,14]. As mentioned in the introduction, this effect has been shown to increase renal sodium reabsorption and potassium excretion, thereby increasing water retention [14]. This in turn brings about the two major side effects that have been associated with GA intake: edema and hypertension [14]. This experiment however suggests no such change in electrolyte balance with the consumption of 100 mg/kg per day GA for four weeks, despite significant reductions in renal 11β-HSD2 levels. This indicates that the major side-effects associated with GA intake would not have occurred, suggesting that suppression of renal 11β-HSD2 activity by GA was not detrimental with the present dosing paradigm. Indeed, we have previously shown that there was no change in blood pressure with GA intake under the same paradigms used in this study [3]. It is hence likely that 11β-HSD2 activities were not sufficiently reduced with the current dosing paradigm, and/or were not reduced for a sufficient duration, to cause an adequate increase in active glucocorticoids, such as would be necessary to cause a hyper-mineralocorticoid effect and subsequent edema and hypertension. It is also important to note that even if edema and hypertension were to occur at some point beyond four weeks, studies show that it would be reversed after GA administration had ceased [23,24,25].

It is possible that the expected increase in the proportion of active glucocorticoids caused by a reduction in renal 11β-HSD2, which would influence parameters affecting blood pressure, may have been countered at least partially by a concomitant reduction in renal 11β-HSD1 activity. Indeed, a previous publication [3] showed a significant drop in renal 11β-HSD1 activity with this same dose and duration of GA administration in rats on a HSD. Moreover, the reductase effects of 11β-HSD1 to activate glucocorticoids is stronger than its dehydrogenase actions to inactivate glucocorticoids in intact cells [12,13], so such a decrease in 11β-HSD1 activity would be expected to shift the balance towards inactive glucocorticoids, which could have countered any adverse effects of reduced 11β-HSD2 activity on blood pressure regulation with the current dosing paradigm. If such a countering effect of reductions in 11β-HSD1 *versus* reductions in 11β-HSD2 activity is occurring at the present dose of GA in rats on a HSD, it is not evident at higher doses of GA, as it is well established that GA, above a certain dosage, indeed brings about edema and hypertension [14].

Another hypothetical explanation for the lack of adverse effects of this dose of GA on electrolyte balance or circulating angiotensin II levels is that the high sucrose diet may be able to block the effects of GA at the level of the mineralocorticoid receptors. While this possibility cannot be excluded at present, we believe it is unlikely because unpublished data from our group indicate that blood pressure remains unchanged with GA administration via the same dosing paradigm in rats that are fed a high fat instead of a high sucrose diet. Moreover, while high sucrose diets *per se* are known to markedly and significantly increase blood pressure in rats [3], this effect is likely mediated by the hyperinsulinemia induced by such diets [3,26] rather than via effects on mineralocorticoid receptors. Indeed, hyperinsulinaemia has been shown to stimulate the RAAS in blood vessels and the heart [27,28], ultimately leading to the overproduction of angiotensin II in these tissues [29]. It has also been shown that high levels of insulin directly enhance the expression and production of angiotensinogen [30], which, with the availability of renin, leads to an increased amount of angiotensin II. Furthermore, hyperinsulinaemia is linked to up-regulation of angiotensin converting enzyme (ACE) activity [31], which is responsible for the conversion of angiotensin I into angiotensin II. The important point from the current study, with respect to the potential safety of orally-administered GA, is that while high sucrose diets lead to increased blood pressure, likely involving effects of hyperinsulinemia, GA—with its known effects on glucocorticoid balance—does not further exacerbate HSD-induced hypertension, when administered with the current dosing paradigm.

Despite the fact that GA administered at the dose and duration of this study did not cause any apparent changes in fluid balance, nor the RAAS, as would be expected during development of side effects, the current GA dosing paradigm was able to maintain metabolic benefits associated with its intake. This dose of GA (100 mg/kg per day, orally administered) has been shown to prevent aberrations in glucose and lipid metabolism caused by a HSD [3]. The current study further extends these findings by showing that GA prevented the HSD-induced increases in adipocyte size and circulating leptin levels without changing caloric intake. Although there was no change in energy intake, the ability of GA to prevent hyperinsulinaemia associated with a HSD, as shown by Chandramouli *et al*. [3], would likely have contributed to the attenuation of leptin levels, as insulin is known to be a major stimulator of leptin secretion [32,33]. Furthermore, there is a link between hyperleptinaemia and adipocyte hypertrophy, and therefore the ability of GA to restrain adipocyte size, as shown in the visceral but not subcutaneous depots in this study, could have also contributed to the observed modulation of leptin levels. This is because leptin biosynthesis increases under conditions of obesity, due in part to enhanced post- and pre-transcriptional effects of insulin and glucocorticoids [34]. The decrease in visceral adipocyte size with GA intake despite no change in energy intake could be attributed at least partially to the decrease in circulating insulin levels, as well as to the increase in lipoprotein lipase (LPL) expression in multiple tissues including the liver, kidney, muscle tissue and white adipose tissue [3]. Such a change could lead to a competitively increased uptake of free fatty acids into non-adipose tissues and a consequentially smaller amount of circulating lipids available for accumulation in adipose tissue [35]. In addition, substances such as thiazolidinediones which can activate peroxisome proliferator-activated receptor γ (PPARγ) in adipose tissue, a capability also shown by GA [3], has been shown to stimulate differentiation of preadipocytes, generating an increased number of smaller adipocytes that are more insulin-sensitive and efficient at storing lipids [36,37,38]. This has been shown to lead to improved insulin sensitivity in the skeletal muscle, probably by reducing circulating free fatty acids and skeletal muscle triglycerides [39]. This may also, in part, be responsible for the reduction of adipocyte size in the visceral depots seen in the current study. PPARγ agonists have also been shown to redistribute fat from visceral to subcutaneous depots, which would explain the lack of a significant difference in subcutaneous adipocyte size despite decreased circulating insulin and increased LPL expression [40].

## 5. Conclusions

Prevalence of the metabolic syndrome has vastly increased over the past 50 years and this in turn is largely responsible for the greater incidence of metabolic diseases such as cardiovascular disease and type II diabetes mellitus. Many of the treatments for the metabolic syndrome today are expensive and/or associated with side effects. When considering the metabolic benefits of GA seen in this experiment and our previous studies [3,9], GA appears to have potential as a viable treatment option for many metabolic disorders associated with the metabolic syndrome, provided that the dosage and duration of treatment is carefully monitored to minimize the known side-effects of fluid imbalance leading to alterations in the RAAS and, subsequently, edema and hypertension. However, it cannot be excluded that edema and hypertension may have eventuated with a longer duration of GA administration, so it would therefore be necessary to understand how long it would take for such side effects to manifest (if at all), by testing longer treatment durations. Furthermore, as it is already understood that stopping GA intake reverses any side effects which have already developed [29,30,31], it would be necessary to study exactly how long such a reversal would take, and also to what extent any metabolic benefits obtained from GA consumption would be maintained after discontinuation. Such knowledge would aid in developing a model for a treatment plan, in order to maximize metabolic benefits and minimize side effects of GA consumption.

## Abbreviations

11β-HSD: 11β-hydroxysteroid dehydrogenase 1
ANOVA: Analysis of variance
EDTA: Ethylenediaminetetraacetate
GA: Glycyrrhizic acid
H & E: Haematoxylin stain and eosin
HPLC: High-performance liquid chromatography
HSD: High-sucrose diet
KRB: Krebs-Ringer bicarbonate
LPL: Lipoprotein lipase
PAS: Periodic acid’s Schiff
PPARγ: Peroxisome proliferator-activated receptor γ
RAAS: Renin angiotensin aldosterone system
SPSS: Statistical package for the social sciences
